# Improving Surgical Innovation: A Cross‐Sectional Survey of Perceived Facilitators and Barriers

**DOI:** 10.1002/wjs.70315

**Published:** 2026-03-10

**Authors:** Clayton R. Baker, Jackson Appelt, Adria A. Villafranca, Carly M. Eckert, Kevin W. Sexton

**Affiliations:** ^1^ Vanderbilt University School of Medicine Nashville Tennessee USA; ^2^ Department of Surgery Vanderbilt University Medical Center Nashville Tennessee USA; ^3^ School of Engineering Duke University Durham North Carolina USA; ^4^ Department of Biomedical Informatics Vanderbilt University Medical Center Nashville Tennessee USA

**Keywords:** implementation, surgical education, surgical innovation

## Abstract

New technologies, processes, or care models that substantially change practice are critical for surgical progress, patient care, and organizational efficiency.

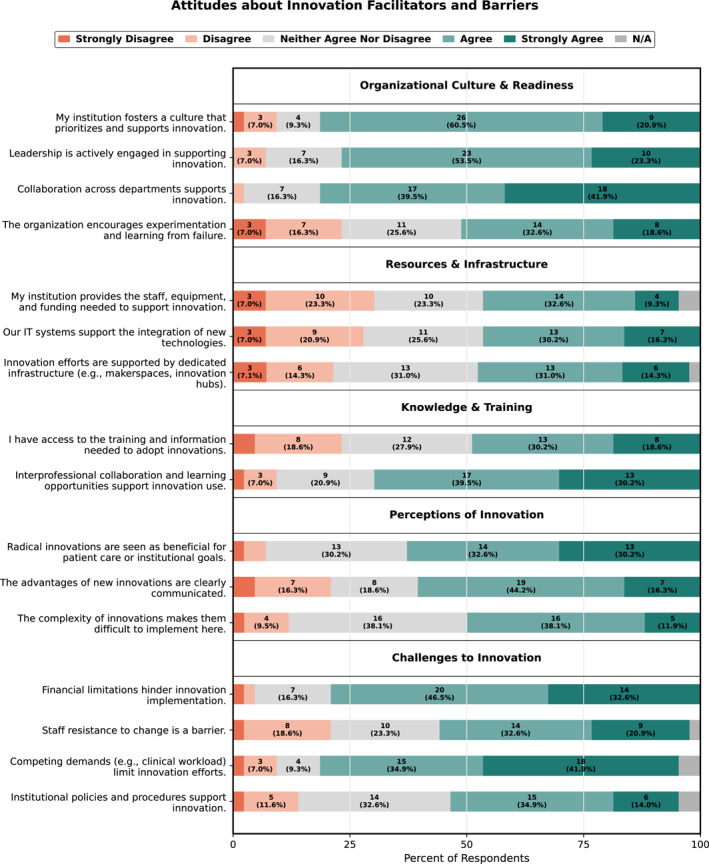

## 1 Introduction

New technologies, processes, or care models that substantially change practice are critical for surgical progress, patient care, and organizational efficiency [1, 2, 3, 4]. Innovation is difficult to implement reliably within complex health systems with entrenched workflows and institutional norms [1, 2, 3, 4]. Development and adoption of innovations often depend on perceived benefit, local fit, and practical environments for testing and refinement [1]. Implementation science frameworks including the Consolidated Framework for Implementation Research (CFIR) emphasize leadership engagement and financial resources as core determinants of routine adoption [5]. In surgery, emerging evidence suggests that successful innovation requires leadership support, operational readiness, accessible training, funding, and protected time [6, 7, 8]. To inform institutional interventions to support these needs, we assessed perceived facilitators and barriers to innovation across surgical faculty, trainees, and advanced practice/clinical support team members.

## 2 Methods

**FIGURE 1:**
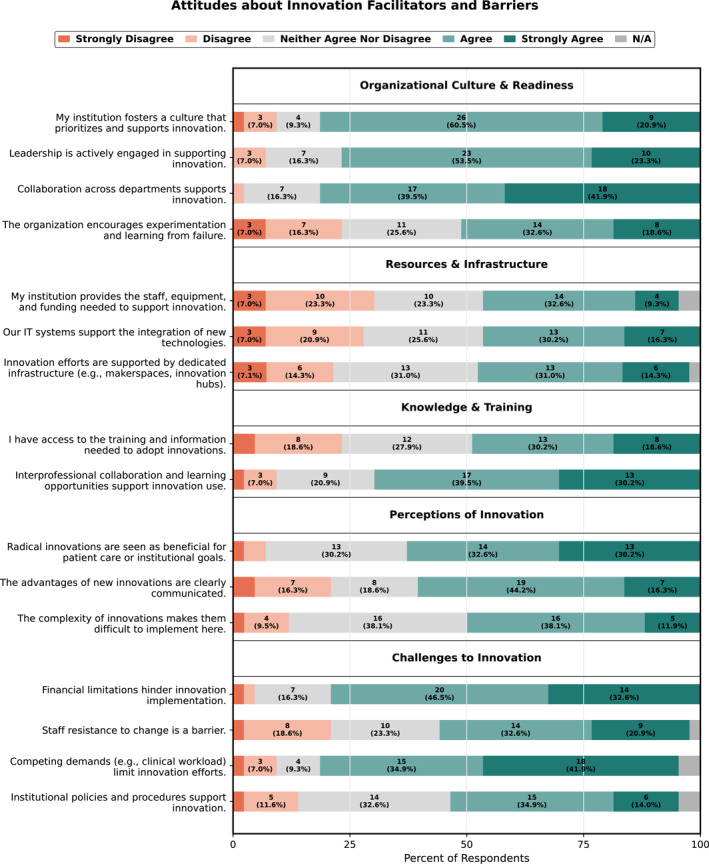
Attitudes about innovation facilitators and barriers.

We conducted a cross‐sectional survey to characterize perceptions of institutional facilitators and barriers to implementing innovation and to identify actionable supports needed to translate innovation into routine practice. The survey—developed in collaboration with our institution's Center for Clinical Quality and Implementation Research—was distributed via institutional listserv to surgical faculty and staff at a single academic institution in December 2025 (IRB#250904). All responses were anonymous, and participation was voluntary. The instrument included 16 statements on a five‐point Likert scale assessing: (1) innovation culture/readiness, (2) resources/infrastructure, (3) training/knowledge accessibility, (4) perceived value, and (5) implementation barriers. All statements are reproduced verbatim in Figure 1. Two free‐response items asked respondents to identify (1) the most significant barrier and (2) the most important strategy for innovation. Recurring themes from free‐text responses were initially generated by a large language model (Microsoft CoPilot). Two authors (CRB, JA) independently reviewed all responses against these categories, iteratively refining and consolidating theme definitions through consensus discussion. Themes were finalized after the consensus coding of all responses. Saturation was confirmed retrospectively when no new themes emerged in the final 20% of responses. Responses could be coded to multiple themes when addressing distinct concepts. Results were presented descriptively and agreement was considered “Agree”/“Strongly Agree” by respondents. Representative quotations were selected by consensus to illustrate themes.

## 3 Results

Of 43 respondents, 61% were male, 44% were attendings, 40% house staff, and 12% advanced practice or support staff. Most respondents agreed their institution prioritizes innovation (81%) and leadership is actively supportive (77%). However, less agreed that their organization encourages experimentation and learning from failure (50%). Collaboration was viewed favorably, with most endorsing cross‐departmental (81%) and interprofessional (70%) collaboration. Most respondents agreed radical innovations benefited patient care or institutional goals (63%) and agreed these advantages were clearly communicated (60%).

Operational readiness was less frequently endorsed; a minority agreed that innovation efforts were supported by dedicated infrastructure (45%), their institution provides adequate staff, equipment, and funding for innovation (42%), or they had information technology (IT) systems to support technology integration (47%). The most frequently endorsed barriers to innovation included financial limitations (79%) and competing demands/workload (77%). By contrast, fewer respondents identified staff resistance to change (48%) or institutional policies and procedures (48%) as major barriers. Complete response distributions are found in Figure 1.

Free‐text items had high completion rates across barrier (88%) and improvement (86%) questions. Respondents described time (39%) and lack of funding (26%) as major barriers, whereas operational/infrastructural support (38%) and funding (30%) were important facilitative strategies (Table 1).

**TABLE 1:** Free‐text themes describing barriers and facilitators to radical innovation implementation, including representative quotations.

What has been the most significant barrier to implementing innovations at your institution? (*n* = 38 responses)		
Theme	*n* (%)	Quotes
Time/workload	15 (39%)	“As a busy clinician who also has to accomplish traditional markers of productivity (ie publication), innovation and entrepreneurship falls behind.”—R11 “My primary barrier is simply time—implementing radical innovation in the time frame of fellowship, while feasible, seems daunting.”—R31
Funding/infrastructure	10 (26%)	“Funding and administrative infrastructure to support innovative initiatives”—R33 “[No] access to innovation start‐up/seed grant programs, especially for young/new investigators to take risks early.”—R28
Mentorship/knowledge/data	9 (24%)	“Lack of knowledge about opportunities,”—R27 “[Lack of] support (financial, time, legal, mentorship).”—R16
Resistance to change/culture	8 (21%)	“…not a culture of addressing obstacles just working around them.”—R44 “…culture of department leaning toward staying with what is comfortable rather than what is innovative or new.”—R39 “Lack of acceptance of new ideas by faculty, staff and administration.”—R25
Legal/regulatory	4 (11%)	“…a slow IRB process…”—R31 “The IRB. They hinder every study, even small ones that are just retrospective reviews. There is no consistency and no help.”—R23
Institutional support	5 (13%)	“Stultifying COI process”—R26 “Ancillary support—there is a very large amount of work that needs to be done (i.e. grants, other paperwork) that's not directly related to innovation.”—R14

What strategies or supports would help facilitate innovation? (*n* = 37 responses)		
Theme	*n* (%)	Quotes
Protected of efficiency in time	7 (19%)	“…Innovation requires protected time and resources.”—R42 “Allowing more support for non‐clinical time.”—R23
Funding/resources	11 (30%)	“There needs to be a dedicated set of funds to support early innovation work. Often times traditional research‐marked funds are not or cannot be dedicated to these larger ideas without clear academic/specialty boundaries.”—R28 “Internal grants, more access to industry to [access] private funding”—R19
Knowledge/training/mentorship	10 (27%)	“Creation of a surgical innovation fellowship.”—R17 “Restructuring the IRB.”—R23 “Additional education to attendings who make decisions for innovations”—R4
Operational/infrastructure/support staff	14 (38%)	“I need to be able to have a conversation about what ideas I have and have someone else do a lot of the leg work.”—R14 “Infrastructure to support development and implementation”—R33
Collaboration/networking	9 (24%)	“Having a forum to discuss ideas and have them evaluated without significant paperwork would be beneficial.”—R14 “Hearing more about innovations happening on campus to know what exists and what is being created to promote more ideas.”—R10
Incentives/culture	8 (22%)	“Promote people who actually have a record of innovation.”—R26 “We are only rewarded with clinical time and effort. If we take away clinical productivity to make room for innovation, we lose revenue. There is no incentive for innovation other than self drive/determination to improve clinical care.”—R23

## 4 Discussion

In this single‐center survey, respondents perceived stronger *cultural* support for surgical innovation but lower *operational* support. This mismatch was reflected in lower endorsement of dedicated infrastructure, resources, and IT integration capacity, alongside high endorsement of time and financial constraints as barriers. Although CFIR is typically applied to evaluate implementation of specific evidence‐based interventions, we adapted it to assess institutional readiness for innovation, revealing that supportive culture alone may be insufficient without operational capacity. Our findings suggest a favorable “inner setting” and implementation climate but limited readiness due to constrained resources. Notably, only half endorsed “experimentation and learning from failure” suggesting low tolerance for setbacks when time and resources are scarce. This pattern mirrors prior literature identifying leadership support and dedicated personnel as facilitators and limited resources and low failure tolerance as barriers [5, 6, 7, 8]. Innovation may be constrained less by resistance to change and more by feasibility limits within existing clinical workloads.

Most respondents recognized the potential benefits of radical innovation for patients and health systems. However, converting endorsement into sustained implementation likely requires practical enablement, translating cultural support into operational pathways that reduce friction. Future strategies include (1) protected time tied to defined implementation milestones and incentives, (2) centralized piloting pathways with clear IT/informatics support, and (3) targeted internal funding with implementation coaching and mentorship.

It remains unclear whether time and funding are primary causal drivers of innovation or early bottlenecks that mask other prerequisites (e.g., mentorship, aligned incentives). Multicenter studies are needed to identify which supports convert resources into durable implementation.

## 5 Limitations

This study is limited by its single‐center design, modest sample size, and descriptive approach without subgroup analysis. Responses may reflect selection and nonresponse bias. Future work should validate findings across multiple institutions and evaluate targeted interventions.

## Author Contributions

**Clayton R. Baker:** conceptualization, methodology, formal analysis, data curation, visualization, writing – original draft, writing – review and editing. **Jackson Appelt:** formal analysis, data curation, writing – original draft, writing – review and editing. **Adria A. Villafranca:** formal analysis, writing – review and editing. **Carly M. Eckert:** conceptualization, writing – review and editing, project administration, supervision. **Kevin W. Sexton:** conceptualization, investigation, funding acquisition, writing – review and editing, methodology, project administration, supervision, resources, data curation.

## Funding

The authors have nothing to report.

## Conflicts of Interest

C.R.B. and J.A. have no disclosures or conflicts of interest.

A.A.V. has equity in Schoolme LLC and hDrop Technologies Inc.

C.M.E. has equity in Schoolme LLC and Avante AI.

K.W.S. has equity in Schoolme LLC; Biometrica Inc.; Arbizal Inc.; hDrop Technologies Inc.; and Qventus Inc. K.W.S. has licensed intellectual property owned by Vanderbilt University Medical Center and the University of Arkansas for Medical Sciences. K.W.S. receives funding from the National Institutes of Health under award numbers R21NR021063 and R01 GM 111324. K.W.S. receives funding from the Advanced Research Projects Agency for Health (ARPA‐H) under project number RSO‐ISO‐5011‐P.

The content is solely the responsibility of the authors and does not necessarily represent the official views of the National Institutes of Health.

## Data Availability

The data that support the findings of this study are available from the corresponding author upon reasonable request.
